# Computational clustering reveals differentiated coronary artery calcium progression at prevalent levels of pulse wave velocity by classifying high-risk patients

**DOI:** 10.3389/fcvm.2023.1161914

**Published:** 2023-05-16

**Authors:** Maximo Rousseau-Portalis, Leandro Cymberknop, Ignacio Farro, Ricardo Armentano

**Affiliations:** ^1^ Bioengineering Research and Development Group, National Technological University, Buenos Aires, Argentina; ^2^ Department of Internal Medicine, Italian Hospital of Buenos Aires, Buenos Aires, Argentina; ^3^ Departamento de Ingeniería Biológica, CENUR Litoral Norte, Universidad de la República, Paysandú, Uruguay

**Keywords:** pulse wave velocity, coronary artery disease, machine learning, clustering, coronary calcium computed tomography

## Abstract

Many studies found that increased arterial stiffness is significantly associated with the presence and progression of Coronary Calcium Score (CCS). However, none so far have used machine learning algorithms to improve their value. Therefore, this study aims to evaluate the association between carotid-femoral Pulse Wave Velocity (cfPWV) and CCS score through computational clustering. We conducted a retrospective cross-sectional study using data from a cardiovascular risk screening program that included 377 participants. We used an unsupervised clustering algorithm using age, weight, height, blood pressure, heart rate, and cfPWV as input variables. Differences between cluster groups were analyzed through Chi-square and T-student tests. The association between (i) cfPWV and age groups, (ii) log (CCS) and age groups, and (iii) cfPWV and log(CCS) were addressed through linear regression analysis. Clusters were labeled *post hoc* based on cardiovascular risk. A “higher-risk group” had significantly higher left (0.76 vs. 0.70 mm, *P* < 0.001) and right (0.71 vs. 0.66 mm, *P* = 0.003) intima-media thickness, CCS (42 vs. 4 Agatston units, *P* = 0.012), and ascending (3.40 vs. 3.20 cm, *P* < 0.001) and descending (2.60 vs. 2.37 cm, *P* < 0.001) aorta diameters. Association with age appeared linear for cfPWV and exponential for log (CCS). The progression of the log (CCS) and cfPWV through age groups was steeper in the “higher-risk group” than in the “lower-risk group”. cfPWV strongly correlated with CCS, and CCS progression over cfPWV differed among clusters. This finding could improve PWV as a “gate-keeper” of CCS testing and potentially enhance cardiovascular risk stratification.

## 1. Introduction

Cardiovascular disease (CVD), majorly through ischemic heart disease, remains the leading cause of death and reduced quality of life worldwide despite enormous advances in controlling blood pressure, diabetes, and hypercholesterolemia (1, 2). As cardiovascular risk stratification is crucial for preventing cardiovascular disease, physicians perform a risk assessment through calculators, such as SCORE, ACVD, WHO, and Framingham, based on traditional cardiovascular risk factors (CVRF) (i.e., sex, age, blood pressure, diabetes, smoking, LDL, HDL) (3).

Although highly useful in clinical practice, these cardiovascular risk estimates are not entirely accurate because traditional CVRFs do not fully explain cardiovascular events. Some studies show that approximately 50% of people do not have any CVRF but still have CVD (4). Moreover, a study showed that about two-thirds of patients undergoing coronary angiography to diagnose CVD had normal coronary arteries (5). Most risk scores have low sensitivity for detecting young individuals with increased absolute cardiovascular risk due to the preponderant effect of age on risk estimation (6, 7). The highest risk for inadequate classification arises mainly in intermediate-risk groups, where CVD prevention is crucial (8, 9).

Individuals with subclinical atherosclerosis have higher cardiovascular events and all-cause death rates (10). In this context, new parameters have been sought to assess cardiovascular risk in search of the variance unexplained by traditional CVRFs and detect subclinical vascular damage. Unlike primary prevention, this strategy permits timely detection of CVD, thus developing more effective and personalized strategies. Novel CVD biomarkers are varied and include biochemical [e.g., C-reactive protein, homocysteine, fibrinogen, lipoprotein(a)], imaging (e.g., coronary artery calcium, intima-media thickness), and biomechanical parameters (e.g., pulse wave velocity, brachial/ankle index, augmentation index) (9, 11–14).

The coronary calcium score (CCS), assessed through a CT scan, is the non-invasive method most commonly used to quantify coronary artery calcifications. CCS has emerged as a robust biomarker of coronary atherosclerosis, can localize lesions, and correlate with plaque burden and cardiovascular disease assessed by angiography (15–18). This method is a strong predictor of cardiovascular events (19) and is considered by ACC/AHA and the European Society of Cardiology for risk reclassification among patients with low to intermediate risk, i.e., a 10-year cardiovascular risk between 6% and 20% (20, 21). Coronary artery calcium scoring has superior discrimination and risk reclassification compared with other subclinical imaging markers or biomarkers (22). Moreover, the absence of coronary calcifications (CCS = 0) has a high negative predictive value for coronary stenosis and, therefore, helps identify individuals at low risk for an event (23). Some cardiovascular risk calculators, such as the MESA, include CCS to perform risk estimations and obtain the vascular age of patients (24). The application of this stratification tool is low despite international recommendations, probably due to its cost and radiation, limiting its availability in low-resource scenarios where cardiovascular risk reclassification of young adults may be needed the most (25).

Pulse wave velocity (PWV) has emerged as a non-invasive, simple, low-cost, reliable, and non-irradiating alternative for assessing subclinical vascular damage (26–28). PWV is a straightforward and tangible way of assessing arterial stiffness—the stiffer the artery, the higher PWV (27). Although there are several ways to measure PWV, carotid-femoral PWV (cfPWV) is the gold standard for assessing large artery stiffness as it has the most clinical evidence of predicting cardiovascular events (26, 27). PWV is a surrogate of CVD as it is a structural and functional marker of cumulative damage to central arteries (27, 29). It is a strong predictor of cardiovascular events (mainly coronary events) independently from traditional CVRFs in both clinical and community-based cohorts (30–33), improving model fit and reclassifying risk for future CVD events in models that include standard risk factors (13). Recently, many studies have used PWV to perform vascular age estimates and divide patients into different phenotypes of vascular aging (34, 35). Despite the existing evidence, there is no unique recommendation regarding PWV's role in risk stratification among international guidelines. In all, most guidelines agree that arterial stiffness may serve as a valuable marker to predict cardiovascular events, but many (especially occidental guidelines) do not recommend systematically measuring PWV in the general population (36).

On the other hand, we are currently witnessing a revolution in cardiology by accumulating large volumes of data from electronic health records, medical imaging data, clinical trials, biobanks, and wearable devices (37). In this regard, medical “Big Data” can nurture Machine Learning (ML, a branch of artificial intelligence) algorithms to differentiate structural and functional patterns embedded in multiple datasets, potentially reduce diagnostic and therapeutic errors, and improve timing, efficiency, and poor workflow (38, 39). Artificial intelligence (AI) has been a late entrant in healthcare (40), although its application has already spurred in many areas, including imaging (37), pathology (41), dermatology (42), and others (38). To date, there is a huge scope in ML algorithms in predicting cardiovascular disease, and there are several AI applications already cleared by the FDA (41).

One of the most used unsupervised learning algorithms is clustering. This ML technique allows subjects to be segregated into distinct groups without prior labeling. These models divide data into groups depending on the “similarity” between data points, which is particularly useful when there are no apparent patterns. Likewise, clustering may contribute to identifying novel biomarkers, disease subgroups, and predictors of clinical outcomes (43). Some studies show clinically relevant and practical applications of these models in cardiovascular medicine (44, 45).

As cardiovascular imaging rapidly grows and has a major cost counterpoint, AI may reduce the financial burden and improve value (37). As with other imaging studies, CCS appears to be an excellent candidate for artificial intelligence tools (46). Although less extensively explored, ML algorithms applied to PWV have demonstrated an improved testing value (47).

Multiple uses for ML algorithms have been proposed in cardiovascular medicine, but none so far have been used to evaluate the association between PWV and CCS. This approach may identify higher-risk individuals who would benefit most from a CCS assessment and avoid unnecessary costs and radiation associated with CT scans in patients with a low risk of presenting coronary disease. Therefore, this study aims to evaluate in a community-based cohort of patients the association between cfPWV and CCS through clustering.

## 2. Methods

### 2.1. Participants

We conducted a retrospective cross-sectional study on participants from a cardiovascular risk screening program that were assessed for demographic, anthropometric, laboratory, and hemodynamic variables related to CVD (48). Participants were included in this study if they had at least one of the following cardiovascular risk factors: advanced age, hypertension, hypercholesterolemia, type two diabetes mellitus, or being a current smoker. Participants were excluded if they lacked assessment of subclinical CVD by cfPWV and CCS and were also excluded those who had a known history of coronary heart disease or stroke.

### 2.2. Variables

The workflow and the gathering and definitions of variables are described in another study (48). Measurements of cfPWV (14) and CCS (49) are also described elsewhere. The cardiovascular risk at 10 years was estimated using the Framingham (50) and SCORE models (51) by entering age, total cholesterol, high-density lipoprotein (HDL) cholesterol, and systolic blood pressure as continuous variables and sex, diabetes, and current smoking as categorical variables (presence or absence).

### 2.3. Clustering

Subsequently, the data underwent cluster analysis. First, the data were normalized, allowing the variables to follow a uniform scale. Second, the principal component analysis technique was applied to reduce dimensionality, and third, the subjects' similarities were calculated in terms of distance measures between variables (Euclidean distance) using the k-means technique. The number of clusters was determined using the Silhouette coefficient. By this technique, we concluded that having two clusters was the most appropriate configuration (52).

The used model requires input variables to assign individuals into groups automatically. In this case, we chose variables relevant for a cardiovascular assessment that could be easily collected in a physician's office, adding the cfPWV. Therefore, age, weight, height, systolic and diastolic blood pressure, heart rate, and cfPWV were included to perform cluster analysis.

### 2.4. Statistical analysis

Numerical variables were summarized using mean and standard deviation for variables with a normal distribution and median and interquartile range for variables with a non-normal distribution. Normality was qualitatively assessed through histograms and formally assessed through the Shapiro-Wilk test. Categorical variables were summarized in percentages in each category. Bivariate analysis between cluster groups regarding categorical and numerical variables were performed using the chi-square test and *T*-student test, respectively. Furthermore, we stratified patients into age groups (<40, 40–49, 50–59, ≥60 years) to see the differences within the variables of interest among clusters and the entire sample and address this potential confounder. Using the mean values of each age group, we performed three exploratory linear regression models, including (i) cfPWV and age groups, (ii) the log transformation of CCS [log (CCS)] and age groups, and (iii) cfPWV and log (CCS). All hypothesis testing involved two-tailed tests with a significance level of *α* = 0.05 and a power of 1-*β* = 80% were performed in the free open-source R program version 4.0.2.

### 2.5. Ethics

The study was conducted following the principles of the Declaration of Helsinki, and the ethics committee of the Bioengineering Research and Development Group of the National Technological University of Buenos Aires, Argentina, approved this study.

## 3. Results

377 individuals from the screening program were finally included in the study. Table 1 describes the overall characteristics of patients and their segregation into clusters. Clusters were labeled after the clustering analysis based on their cardiovascular risk differences through Framingham and SCORE in a “higher-risk group” and a “lower-risk group”. Regarding output variables only, we found that the “higher-risk group” had significantly higher left (0.76 vs. 0.70 mm, *P* < 0.001) and right (0.71 vs. 0.66 mm, *P* = 0.003) IMT, CCS (42 vs. 4 Agatston units, *P* = 0.012), ascending (3.40 vs. 3.20 cm, *P* < 0.001) and descending (2.60 vs. 2.37 cm, *P* < 0.001) aorta diameters, and Framingham (*P* < 0.001) and SCORE (*P* = 0.003) 10-year risk estimators than the “lower-risk group”.

**Table 1:** General characteristics and segregation of individuals through clustering^a^.

Variables	General characteristics (*N* = 377)	Cluster groups^b^		*P*-value
		“Lower-risk group” (*N* = 214)	“Higher-risk group” (*N* = 163)	
Demographics and anthropometrics				
Age (years)	56.5 ± 9.11	54.3 ± 8.61	59.3 ± 9.00	<0.001
Female sex—*n* (%)	115 (30.5)	74 (34.6)	41 (25.2)	0.063
Body mass index (kg/m^2^)	26.1 ± 4.27	25.1 ± 4.07	27.4 ± 4.20	<0.001
Medical history				
Arterial hypertension—*n* (%)	185 (49.1)	67 (31.3)	118 (72.4)	<0.001
Hypercholesterolemia—*n* (%)	307 (81.4)	174 (81.3)	133 (81.6)	1
Diabetes—*n* (%)	24 (6.4)	11 (5.1)	13 (8.0)	0.366
Smoking—*n* (%)	83 (21.5)	50 (23.4)	30 (18.4)	0.298
Presence of carotid plaque—*n* (%)	199 (52.8)	108 (50.5)	91 (55.8)	0.353
Presence of femoral plaque—*n* (%)	250 (66.3)	134 (62.6)	116 (71.2)	0.103
Hemodynamics				
Systolic blood pressure (mmHg)	124 ± 13.3	116 ± 8.25	135 ± 10.8	<0.001
Diastolic blood pressure (mmHg)	73.3 ± 9.43	68.5 ± 7.29	79.6 ± 8.14	<0.001
Heart rate (bpm)	65.5 ± 9.99	63.5 ± 9.62	68.1 ± 9.89	<0.001
IMT from right carotid artery (mm)	0.68 ± 0.13	0.66 ± 0.13	0.71 ± 0.13	0.003
IMT from left carotid artery (mm)	0.72 ± 0.147	0.70 ± 0.13	0.76 ± 0.16	<0.001
cfPWV (m/s)	10.6 ± 2.70	9.46 ± 1.77	12.2 ± 2.92	<0.001
CCS (Agatston units)	14.0 [0, 131]	4.50 [0, 80.8]	42.0 [0, 209]	0.012
Ascending aorta diameter (cm)	3.29 ± 0.39	3.20 ± 0.37	3.40 ± 0.39	<0.001
Descending aorta diameter (cm)	2.47 ± 0.26	2.37 ± 0.24	2.60 ± 0.22	<0.001
Cardiovascular risk				
Framingham—*n* (%)				
<10%	143 (37.9)	116 (54.2)	27 (16.6)	<0.001
10%–20%	159 (42.2)	83 (38.8)	76 (46.6)	
>20%	75 (19.9)	15 (7.0)	60 (36.8)	
SCORE—*n* (%)				
0%	233 (61.8)	147 (68.7)	86 (52.8)	0.004
1%	105 (27.9)	51 (23.8)	54 (33.1)	
2%	21 (5.6)	11 (5.1)	10 (6.1)	
3%–4%	15 (4.0)	3 (1.4)	12 (7.4)	
5%–9%	3 (0.8)	2 (0.9)	1 (0.6)	

CCS is expressed as median and interquartile range, following a non-normal distribution. *P* < 0.05 was considered statistically significant. Hb1Ac, glycolized hemoglobin; HDL, high-density lipoprotein; LDL, high-density lipoprotein; IMT, intima-media thickness; cfPWV, carotid-femoral pulse wave velocity; CCS, coronary calcium score.

^a^ Plus-minus values are mean ± standard deviation.

^b^ Cluster groups were labeled after the clustering analysis.

To weigh the benefit of including arterial stiffness in the model, we performed a sensitivity analysis without cfPWV as an input variable. We found that the latter model underperformed the original model regarding CCS, IMT, and cardiovascular risk discrimination (see Supplementary Material S1).

We found a significant difference in cfPWV and CCS among age groups, where cfPWV and CCS increased with age in the “lower-risk group”, the “higher-risk group”, and the entire sample (see Supplementary Material S2).

**Figure 1:**
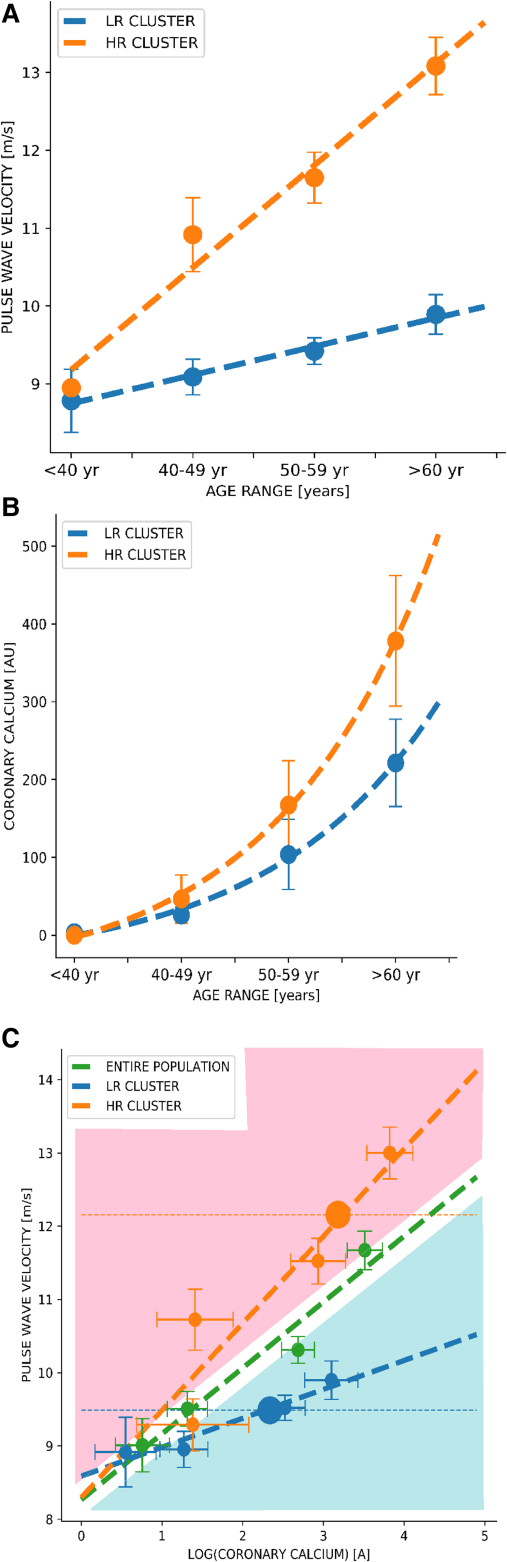
Dynamics of pulse wave velocity vs. coronary artery calcium curves through clustering among age groups. (**A**) Correlation between Pulse Wave Velocity and age groups. (**B**) Correlation between Coronary Calcium Score and age groups. (**C**) Correlation between Pulse Wave Velocity and Coronary Calcium Score. Green curves correspond to the analysis of the entire population, and blue and orange curves correspond to the lower-risk and higher-risk groups, respectively. Values are shown as the mean (dots) and 95% confidence interval (bars). A, Agatston units; m/s, meters per second; yr, years; LR cluster, lower-risk cluster; HR cluster, higher-risk cluster.

Through a linear regression, we found that the progression of PWV and CCS over age differed significantly among clusters, being steeper for the “higher-risk group” compared with the “lower-risk group”. This association with age appeared as linear for cfPWV (Figure 1A) and exponential (Figure 1B) for log (CCS). We also found that the association between cfPWV and CCS differed significantly between clusters, where the linear trends in PWV and CCS progression over age change depending on the assigned cluster group. The progression of the log (CCS) and cfPWV through age groups was steeper in the “higher-risk group” than in the “lower-risk group”. Moreover, we found that the cfPWV of the cluster centroid belonging to the “higher-risk group” was 12 m/s compared to 9.5 m/s from the centroid belonging to the “lower-risk group” (Figure 1C).

## 4. Discussion

In a sample of adults included in a cardiovascular screening program, we found that a clustering algorithm—only using age, weight, height, systolic and diastolic blood pressure, heart rate, and cfPWV as input variables—separated the cohort into two differentiated groups, in terms of traditional cardiovascular risk factor scores (i.e., Framingham, SCORE) and subclinical cardiovascular disease parameters. Basically, one group showed higher cardiovascular risk, greater intima-media thickness, higher CCS score, greater aorta diameter, and, as expected, higher cfPWV (consequently named “higher-risk group”). Moreover, CCS progression over cfPWV differed among clusters. The “higher-risk group” had a steeper trend in the progression of cfPWV and CCS over age and cfPWV over CCS compared to the cluster named “lower-risk group” (Figure 1C). We also found that the cfPWV of the cluster centroid belonging to the “higher-risk group” was 12 m/s compared to 9.5 m/s from the centroid belonging to the “lower-risk group”. Concerning the latter, it is interesting to point out that the Korean (53) and Japanese (54) guidelines consider a cfPWV >10 m/s as an indicator of subclinical organ damage.

Our first finding is that cfPWV correlated well with CCS. Kim et al. (26) have broadly reviewed in their paper on “*Pulse Wave Velocity in Atherosclerosis*” the body of evidence exploring the association between PWV and CCS as a surrogate of coronary atherosclerosis. Studies found that increased arterial stiffness is significantly associated with both the presence and progression of CCS. This association has been replicated in community-based cohorts undergoing health check-ups (55–59), patients with type two diabetes (60, 61), and with suspected coronary artery disease undergoing coronary angiography (62). Despite cfPWV being the gold standard, most studies have used baPWV, probably due to the preponderance of Asian evidence, where baPWV is most commonly used (63). Regardless of demonstrating causality—which is challenging due to scarce longitudinal studies (26, 55)—the proxy for this finding is identifying individuals with increased arterial stiffness and thereby increased likelihood of presenting coronary calcifications. In this way, PWV may be a “gate-keeper” for CCS testing and reduce the costs and radiation associated with CCS measurement (27). Our results align with current evidence concerning the strong association between PWV and coronary atherosclerosis. However, our novel approach suggests that running the data through clustering reveals two different laws in the association of PWV and CCS.

Arterial stiffness is one of the earliest markers of atherosclerosis and vascular aging (29, 64) and can be accelerated by other cardiovascular risk factors (65). From a physiopathology standpoint, atherosclerosis and arteriosclerosis appear to be the underlying processes behind CCS and PWV changes, respectively. There are common risk factors (e.g., hypertension, dyslipidemia, diabetes, smoking) among them, and their pathologic mechanisms appear to overlap (66). Arterial stiffness involves changes in the extracellular matrix, including elastin degradation and collagen deposition, leading to atherosclerosis (65). Vascular remodeling in the presence of increased stiffness could predispose to calcification of the intima and media layer (67) and increased wall shear stress and afterload hence triggering a pathophysiological cascade leading to atherosclerosis and plaque rupture (68) and cardiac remodeling (27, 55). Moreover, arterial stiffness reduces the buffering in the aortic trunk that helps maintain coronary flow during diastole, further limiting coronary perfusion in an occluded lumen (69). In turn, atherosclerosis generates endothelial dysfunction and structural wall changes, increasing arterial stiffness (70).

We are currently seeing a paradigm shift from population-based care into the precision medicine era (39). AI, through ML methods, could help achieve a “personalized” medicine by summarizing enormous quantities of medical information rather than a rigidly defined “one-size-fits-all” algorithm commonly seen in everyday physician practice (38). The advantages of AI tools include freedom from statistical assumptions, exploring multiple hidden patterns, and learning independently (71, 72). These features could revolutionize healthcare and offer the potential for earlier detection of disease, improved diagnostic accuracy, and more accurate prognosis and disease severity prediction to guide optimal management (73, 74).

Although this is the first time cfPWV and CCS association using ML algorithms has been evaluated, there are applications already used for these parameters individually. Automatic and semi-automatic AI-based methods correlate robustly with traditional CCS computation methods but consume less time and resources (75). For example, Motwani et al. developed an ML model that outperformed the Framingham risk score and computed tomography coronary calcification angiography (CTCA) severity risk scores to predict 5-year all-cause mortality in patients who underwent CTCA (76). Also, “Zebra Medical Vision” has recently acquired an FDA clearance for an AI-based tool to assess cardiovascular risk in patients based on coronary artery calcium (77, 78). As for PWV, Vallée et al. found the PWV index as the main factor for predicting coronary heart disease through decision tree models (79). Also, they created artificial neural network models, which included the PWV index that accurately predicted coronary heart disease (80).

By having access to the machine learning algorithm used in this study and cfPWV assessment, one could automatically determine the patient cluster assignment. However, for cases where individual patients are not included in the algorithm, the complementary method for clinical use is proposed:

Physicians could manually determine cluster assignment using patients' cfPWV and cfPWV vs. age curves generated by the system (Figure 1A). Given the patient's age and cfPWV values, we can fix—as in a nomogram—where the point falls and thus graphically assign a cluster without necessarily including the patient in the system. If cfPWV value falls below the curve corresponding to the general population (blue area), the patient could be assigned to the “lower-risk group”, and if it falls upwards (red area), it could be assigned to the “higher-risk group”. Subsequently, we could estimate the patients' PWV progression based on the assigned cluster and graphically estimate patients' coronary calcium based on the assigned cluster and on the log (CCS) vs. age curves (Figure 1B). Thus, considering the patient's comorbidities, lifestyle, and measurement of cfPWV, physicians can use CCS prediction curves and—depending on the clustering group assignment—evaluate the necessity of ratifying calcium values through a CT scan. Through this clustering algorithm, we could generate new curves every year by analyzing different populations with cfPWV and CCS measurements to improve stratification.

The proposed method may enhance the usefulness of subclinical vascular damage screening tools and aid the commonly challenging decision of starting pharmacological therapy (i.e., statins). It should be mentioned that the cardiovascular risk assessment carried out in this study does not aim to replace traditional risk scores, but instead be a complement in scenarios where PVW and (eventually) CCS might be available. Therefore, mid-age patients who have already been evaluated by multivariate risk models such as SCORE or Framingham and have borderline 10-year cardiovascular risk (81) or young individuals where risk scores underestimate cardiovascular risk and need direct subclinical disease assessment might benefit from this proposal (82).

Despite our study findings, we must acknowledge its limitations. First, inclusion criteria and clustering models were generated using retrospective data from a single center, limiting the external validity of results to other areas. Secondly, machine learning algorithms nurture themselves from Big Data and most often need an enormous quantity of info to take advantage of its usefulness. In this study, we only had a small quantity of data in terms of model generation, which increases the uncertainty of the trends shown. Third, clustering algorithms are dependent on the initial cluster pattern. Therefore, the method should be reproduced in other cohorts to address this potential bias to archive robust conclusions about this methodology (83).

Applying this ML tool may extend the usage of PWV and CCS in clinical practice and help reduce the global burden of CVD. Nevertheless, these preliminary findings warrant validation by more extensive and heterogeneous patient cohorts to elucidate the benefit of clustering and explore the benefits of other ML algorithms in PWV and CCS association.

## 5. Conclusions

This paper sought to illustrate the state-of-the-art of two subclinical vascular damage parameters and how their use may be enhanced through ML goggles. We found that a clustering algorithm segregated CCS progression over cfPWV into two groups of patients with divergent subclinical vascular disease. By these findings, we intend to identify a higher-risk cluster with a higher probability of detecting coronary artery disease by CCS and saving cost and radiation in patients belonging to a lower-risk cluster. In all, this finding could improve PWV as a “gate-keeper” of CCS testing and potentially enhance patient stratification.

## Data Availability

The data analyzed in this study is subject to the following licenses/restrictions: If necessary datasets and results may be provided to confirm results. Requests to access these datasets should be directed to Maximo Rousseau-Portalis, mrousseauportalis@frba.utn.edu.ar.
