# Supplementary material for: Computational clustering reveals differentiated coronary artery calcium progression at prevalent levels of pulse wave velocity by classifying high-risk patients
Source: Front Cardiovasc Med. 2023 May 16;10:1161914. doi: 10.3389/fcvm.2023.1161914 (PMC10228741; doi:10.3389/fcvm.2023.1161914)
Supplement: Supplementary file 1 [file Table1.docx]

| Clustering **without** cfPWV input  N_1_=223 vs N_2_=154 |
| --- |
| **Framingham:** 11% vs 15%; p <0.001 |
| **SCORE:** 1.27% vs 1.29% ; p >0.05 |
| **cfPWV***  10.7 (2.5) vs 10.5 (2.9) m/s; p >0.05 |
| **Right IMT***  0.67 (0.2) vs 0.68 (0.1) mm; p >0.05 |
| **Carotid plaques**  117 (52%) vs 82 (53%); p >0.05 |
| **Coronary Calcium Score** ±  13 [0, 122] vs 14.5 [0, 132] UA; p >0.05 |

| Clustering **with** cfPWV input  N_1_=214 vs N_2_=163 |
| --- |
| **Framingham:** 10% vs 18%; p <0.001 |
| **SCORE:** 1.08% vs 1.56%; p <0.001 |
| **cfPWV***  9.5 (1.8) vs 12.2 (2.9) m/s; p <0.001 |
| **Right IMT***  0.66 (0.1) vs 0.70 (0.1) mm; p <0.05 |
| **Carotid plaques**  108 (51%) vs 91 (56%); p >0.05 |
| **Coronary Calcium Score** ±  4.5 [0, 81] vs 42.0 [0, 209] AU; p <0.05 |

*Expressed as mean (standard deviation)

± Expressed as median [interquartile range]

Abbreviations: cfPWV: carotid-femoral pulse wave velocity. IMT: Intima Media Thickness.
